# Mechanism of Guilu Erxian ointment based on targeted metabolomics in intervening *in vitro* fertilization and embryo transfer outcome in older patients with poor ovarian response of kidney-qi deficiency type

**DOI:** 10.3389/fendo.2023.1045384

**Published:** 2023-01-20

**Authors:** Yingjie Ma, Jingyan Song, Xianling Cao, Zhengao Sun

**Affiliations:** ^1^ Shandong University of Traditional Chinese Medicine, First Clinical Medical College, Jinan, China; ^2^ Integrative Medicine Research Centre of Reproduction and Heredity, The Affiliated Hospital of Shandong University of Traditional Chinese Medicine, Jinan, China

**Keywords:** targeted metabolomics, advanced age and low response, kidney qi, metabolomics, POR

## Abstract

**Objective:**

To study the effect of Guilu Erxian ointment on the outcome of IVF-ET in older patients with poor ovarian response infertility of kidney-qi deficiency type, and to verify and analyze the mechanism of action of traditional Chinese medicine on improving older patients with poor ovarian response infertility of kidney-qi deficiency type from the perspective of metabolomics using targeted metabolomics technology, identify the related metabolic pathways, and provide metabolic biomarker basis and clinical treatment ideas for improving older patients with poor ovarian response infertility.

**Methods:**

This study was a double-blind, randomized, placebo-controlled trial, and a total of 119 infertile patients who underwent IVF-ET at Shandong Center for Reproduction and Genetics of Integrated Traditional Chinese and Western Medicine were selected. Eighty older patients with infertility undergoing IVF were randomly divided into older treatment group and older placebo group, and another 39 young healthy women who underwent IVF-ET or ICSI due to male factors were selected as the normal control group. Flexible GnRH antagonist protocol was used for ovulation induction in all three groups, and Guilu Erxian ointment and placebo groups started taking Guilu Erxian ointment and placebo from the third day of menstruation until IVF surgery. And ultra-high performance liquid chromatography-triple quadrupole mass spectrometer (UHPLC-QTRAP MS) was used to detect metabolites in the three groups of samples.

**Results:**

Compared with the placebo group, the number of oocytes retrieved, 2PN fertilization, high-quality embryos, total number of available embryos and estrogen on HCG day were increased in the treatment group, and the differences were statistically significant (*P* > 0.05), but the clinical pregnancy rate of fresh embryos and frozen embryos were not statistically significant (*P* > 0.05). The results of targeted metabolomics analysis showed that follicular fluid in the treatment group clustered with the normal young group and deviated from the placebo group. A total of 55 significant differential metabolites were found in the follicular fluid of older patients with poor ovarian response of kidney-qi deficiency type and patients in the normal young group, after Guilu Erxian ointment intervention, Metabolites such as L-Aspartic acid, Glycine, L-Serine, Palmitoleic Acid, Palmitelaidic acid, L-Alanine, Gamma-Linolenic acid, Alpha-Linolenic Acid, and N-acetyltryptophan were down-regulated, mainly involving amino acid metabolism and fatty acid metabolism.

**Conclusion:**

Guilu Erxian ointment can effectively improve the clinical symptoms and IVF outcomes of older patients with poor ovarian response of kidney-qi deficiency type. There were differences in follicular fluid metabolites between older patients with poor ovarian response of kidney-qi deficiency type and normal women. L-Aspartic acid, L-Alanine, Aminoadipic acid, L-Asparagine, L-Arginine, L-Serine, Gamma- Linolenic acid, Pentadecanoic acid and Alpha-Linolenic Acid are closely related to older patients with poor ovarian response due to deficiency of kidney-qi and may be inferred as biomarkers. The mechanism of Guilu Erxian ointment intervention may be mainly through amino acid metabolism and fatty acid metabolism regulation.

The trial design has been published in Trials, https://doi.org/10.1186/s13063-021-05867-5

## 1 Introduction

With increasing age, the reproductive capacity of women gradually decreases more likely due to irreversible ovarian aging leading to diminished ovarian reserve (DOR), but *in vitro* fertilization-embryo transfer (IVF-ET) technology is becoming more and more mature, bringing good news to many older infertile couples (1). Although IVF-ET is rapidly becoming more and more widely used as an effective treatment for infertile couples, it is still a difficult and hot issue for the reproductive boundary to improve the clinical pregnancy rate and live birth rate of older infertile patients, especially for women with poor ovarian response to exogenous gonadotropin stimulation (DOR or POR) (2). Metabolomics has become an increasingly powerful research tool in natural science and life sciences, not only for the overall, systematic, and dynamic analysis of endogenous markers of living organisms, but also for elucidating biological perturbations to internal or external stimuli, which can visually reflect the current metabolic status of organisms or cells (3). In recent years, metabolomics analysis methods have been widely used due to their great advantages compared with nuclear magnetic resonance, GC-MS and other technical means, and metabolomics is to discover potential biomarkers of the disease, explore the pathogenesis of the disease, and provide the possibility for the diagnosis, treatment and prognosis of the disease (4, 5).

With the deepening of traditional Chinese medicine (TCM) in IVF-ET technology, traditional Chinese medicine has a significant effect in improving oocyte quality, improving endometrial receptivity, increasing clinical pregnancy rate, and reducing complications. Traditional Chinese medicine believes that the main causes of infertility in older women are deficiency of kidney qi and deficiency of kidney essence. In view of this symptom, the method of tonifying kidney and supplementing qi can be used to improve infertility in older women. This theory is consistent with relevant western medicine studies. In 2013, the American Association for Assisted Pregnancy counted 467 reproductive clinics (a total of 190773 cycles), and the data showed that the clinical pregnancy failure rate of women would increase greatly at older age, especially after the age of 35 years; The pregnancy failure rate was about 10% in women aged 35 years and could reach 65% by the age of 45 years; The successful pregnancy rate of IVF-ET was close to 50% in women aged 35 years, while it may be less than 10% in women aged 42 years (6).

## 2 Materials and methods

The study protocol was conducted with reference to our team ‘s published protocol (7).

Inclusion criteria: refer to Bologna and Poseidon criteria (8, 9), (1) Age between 35 and 42 years; (2)AFC< 5-7; (3) AMH< 1.1 ng/mL; (4) ≤ 3 oocytes obtained after standard ovarian stimulation; (1) is a necessary condition, and the other three meet one of the conditions. Exclusion criteria: (1)BMI ≥ 25Kg/m^2^; (2)suffering from genetic diseases not suitable for fertility as specified in the Maternal and Child Health Care Law; (3) severe endometriosis, adenomyosis, immune infertility; (4)untreated tubal effusion; (5)congenital or acquired uterine dysplasia and other serious reproductive organ malformations; (6) combined reproductive system tumors, thyroid dysfunction, hyperprolactinemia and other endocrine dysfunction history; (7) received ovarian stimulation therapy or OCP in the past three months; (8) previous ovarian and other gynecological surgery history; (9)and other contraindications to assisted reproductive technology.

### 2.1 Therapeutic drugs

Tortoise shell glue, turtle shell glue, staghorn glue, wolfberry fruit, panax quinquefolium, cornus officinalis, hawthorn seed, jujube. Preparation method: It was provided by Hunan Lao TCM Pharmaceutical Co., Ltd., and the extraction step was based on the SOP of the preparation room. Method of administration: 300 ml per bottle, orally, 5 ml each time, 3 times a day, Guilu Erxian ointment was administered on the third day of menstruation in the menstrual cycle before IVF for 40 days.

### 2.2 Placebo

Placebo is provided by Hunan Lao TCM Pharmaceutical Co., Ltd., which can simulate the appearance, color and odor of the formula using new technology, but has no active ingredients and therefore has no efficacy. Placebo plaster is indistinguishable from Guilu Erxian plaster in appearance and taste. The method of administration was the same as Guilu Erxian Ointment.

### 2.3 Ovarian stimulation and oocyte retrieval

Ovarian stimulation began on the second day of menstruation, and all patients were given recombinant follicle stimulating hormone (r-FSH) (Gonal-F, Merck Serono, Switzerland) or urinary gonadotropins. The initial FSH dose is determined by the physician based on age, antral follicle count (AFC), basal FSH, estradiol (E_2_) levels, and body mass index (BMI), which typically ranges from 150 to 300 U/day. This dose was adjusted every 2 to 3 days depending on ovarian response, E_2_ level, and follicular growth under ultrasound monitoring. Then, all patients were treated with GnRH-ant from day 6 of COH or when the dominant follicle reached 14 mm in diameter until the day before trigger. Finally, when the mean diameter of ≥ 3 follicles reached 17 mm, 8000 to 10000 U of urine-derived hCG or 250 μg of r-hCG was used. Thirty-five to 36 hours after r-hCG injection, oocyte retrieval was guided by transvaginal ultrasound. The first follicular fluid was obtained from the patient, which was required to be bloodless, clear, and yellowish, and the follicular fluid was confirmed to have oocyte cumulus coronavirus complex (QCCC) by microscopy, 3000 r/min, centrifuged for 10 min, and the supernatant was loaded into Ependorf (EP). Intracytoplasmic sperm injection (ICSI) is performed only in cases of severe male factor infertility.

### 2.4 Embryo transfer protocol

According to the patient ‘s endometrium, P value, embryo score and personal requirements, it is decided whether fresh or frozen embryo transfer should be performed, such as embryo transfer on the third day after oocyte retrieval, with the following exceptions: (1) serum E_2_ > 5000 pg/mL on the trigger day; (2) number of oocytes retrieved ≥ 15; (3) OHSS; (4) uterine or endometrial abnormalities, such as endometriosis, uterine fibroids, endometrial polyps or intrauterine adhesions; (5) serum P > 1.75 ng/mL on the trigger day. No more than 2 embryos were transferred per transfer. Luteal support was started on the first day after oocyte retrieval, and progesterone 40 mg/day or progesterone vaginal sustained-release gel 90 mg/day was intramuscularly injected using luteal support after transplantation, along with dydrogesterone 20 mg/day, from the day of transplantation until 12 weeks of gestation.

### 2.5 Sample collection and management

Follicular fluid with mature oocytes was collected within 1 hour after oocyte retrieval, centrifuged on a 3000 g centrifuge for 10 minutes, and the yellowish, bloodless, clear supernatant was loaded into Epppendorf tubes and then cryopreserved in a -80°C freezer at our reproductive center.

### 2.6 Experimental instruments and reagents

AB 5500/6500 Q-trap mass spectrometer (AB SCIEX); Agilent 1290 Infinity LC ultra-high pressure liquid chromatograph (Agilent); low temperature high speed centrifuge (Eppendorf 5430R); Chromatographic column: Waters, ACQUITY UPLC BEH Amide 1.7μm, 2.1mm × 100 mm Waters, ACQUITY UPLC BEH C18 1.7μm, 2.1mm × 100mm column; Acetonitrile (Merck, 1499230-935); Ammonium acetate (Sigma, 70221); Methanol (Fisher, A456-4); ammonia (Sigma, 221228); Ammonium formate (Sigma, 70221); Formic acid (Sigma, 00940); Isotope standard (Isotope Laboratories).

### 2.7 Sample extraction method

After the sample was slowly thawed at 4°C, an appropriate amount of the sample was added with precooled methanol/acetonitrile/water solution (2:2:1, v/v), vortex mixed, sonicated at low temperature for 30 min, allowed to stand at -20°C for 10 min, centrifuged at 14,000 g for 20 min at 4°C, the supernatant was vacuum-dried, and 100 μL of acetonitrile aqueous solution (acetonitrile: water = 1:1, v/v) was added for reconstitution during mass spectrometry, vortex, centrifuged at 14000 g for 15 min at 4°C, and the supernatant was injected for analysis.

### 2.8 Chromatography-mass spectrometry

Samples were separated on Agilent 1290 Infinity LC Ultra Performance Liquid Chromatography (UHPLC) HILIC and C18 columns; HILIC column temperature 35°C; flow rate 0.3 mL/min; injection volume 2 μL; mobile phase composition A: 90% water + 2 mM ammonium formate + 10% acetonitrile, B; methanol + 0.4% formic acid; gradient elution program was as follows: 0-1.0 min, 85% B; 1.0-3.0 min, B changed linearly from 85% to 80%; 3.0-4.0 min, 80% B; 4.0-6.0 min, B changed linearly from 80% to 70%; 6.0-10.0 min, B changed linearly from 70% to 50%; 10-12.5 min, B maintained at 50%; 12.5-12.6 min, B changed linearly from 50% to 85%; 12.6-18 min, B maintained at 85%. C18 column temperature 40°C; flow rate 0.4mL/min; injection volume 2μL; mobile phase composition A: water + 5 mM ammonium acetate + 0.2% ammonia, B: 99.5%; acetonitrile + 0.5% ammonia; gradient elution program was as follows: 0-5 min, B changed linearly from 5% to 60%; 5-11 min, B changed linearly from 60% to 100%; 11-13 min, B maintained at 100%; 13-13.1 min, B changed linearly from 100% to 5%; 13.1-16 min, B maintained at 5%; samples were placed in an autosampler at 4°C throughout the analysis. In order to avoid the influence caused by the fluctuation of signal detected by the instrument, continuous analysis of samples was performed in random order. QC samples were inserted into the sample cohort to monitor and evaluate the stability of the system and the reliability of the experimental data. Mass spectrometry was performed using an AB 6500 QTRAP mass spectrometer (AB SCIEX). The ESI source conditions were as follows: sheath gas temperature, 350°C; dry gas temperature, 350°C; sheath gas flow, 11 L/min; dry gas flow, 10L/min; capillary voltage, 4000 V or -3500 V in positive or negative voltage, modes; nozzle, 500 V; and nebulizer pressure, 30 psi, monitored using MRM mode.

### 2.9 Statistical analysis

All data are reported as mean ± standard deviation, For baseline comparisons, differences in means of continuous data between placebo and intervention groups will be evaluated using independent Student t test or Mann-Whitney U test depending on whether the data are normally distributed, and frequencies will be compared using Chi-square test. For longitudinal data with baseline, we will evaluate mean differences between and within groups using repeated measures analysis of covariance (ANCOVA). All tests will be two-tailed and Fisher ‘s exact rate method will be used to compare ratios between the two groups, with significance level defined as a value of *P*< 0.05.

## 3 Study results

### 3.1 Clinical outcome

#### 3.1.1 Comparison of basic data among three groups

There was no significant difference in basic data between the treatment group and placebo (*P* > 0.05), which was comparable. The age, gravidity, parity, proportion of secondary infertility and basal FSH in the treatment group and placebo group were higher than those in the normal control group, and the differences were statistically significant (*P* > 0.05). The anti-Müllerianhormone (AMH) in the normal control group was higher than that in the treatment group and placebo group, and the differences were statistically significant (*P* > 0.01). See **Table 1** for details

**Table 1:** Comparison of basic data among three groups [( $\bar{x}$ ± s),%].

Basic data	Treatment group (N=38);	Placebo group (N=37);	*P*1 value	Normal group (N=39);	*P*2 value
Age (years)	36.87 ± 3.12	37.49 ± 3.19	0.379	29.23 ± 2.79	0.001
Duration of infertility (years)	3.18 ± 2.36	3.11 ± 1.88	0.454	3.10 ± 1.59	0.983
BMI (kg/m ^2^)	24.73 ± 4.24	23.34 ± 2.46	0.207	23.04 ± 6.51	0.256
G (Gravidity)	1.26 ± 1.00	1.27 ± 0.96	0.975	0.72 ± 0.94	0.019
P (Parity)	0.50 ± 0.51	0.51 ± 0.61	0.905	0.10 ± 0.31	0.001
A (Abortions)	0.63 ± 0.75	0.62 ± 0.68	0.952	0.44 ± 0.72	0.406
Secondary infertility ratio	76.31% (29/38)	72.97% (27/37)	0.739	46.15% (18/39)	0.007
AMH (ng/mL)	0.96 ± 0.53	0.97 ± 0.73	0.977	6.86 ± 4.56	0.001
Basal FSH (mIU/mL)	9.92 ± 2.17	10.90 ± 7.20	0.329	6.44 ± 1.54	0.001
Basal LH (mIU/mL)	4.43 ± 1.59	4.69 ± 3.15	0.623	5.25 ± 1.59	0.086
Basal E_2_ (pg/mL)	46.13 ± 13.28	47.02 ± 15.65	0.852	46.60 ± 29.09	0.983
Basal P (ng/mL)	0.70 ± 0.55	0.74 ± 0.58	0.775	0.58 ± 0.55	0.442

P1 value is the treatment group versus placebo group; P2 value is the comparison among three groups

#### 3.1.2 Ovarian stimulation comparison

E_2_ on HCG day in the treatment group was higher than that in the placebo group, and the difference was statistically significant (*P* > 0.01). There was no significant difference in Gn days, total Gn and HCG day (*P* > 0.05). The Gn days and total Gn in the normal group were lower than those in the treatment group and placebo group, and the difference was statistically significant (*P* > 0.01). E_2_ on HCG day in the normal group was higher than that in the treatment group and placebo group, and the difference was statistically significant (*P* > 0.01). P on HCG day in the normal group was not statistically significant compared with the other two groups (*P* > 0.05). See **Table 2** for details

**Table 2:** Comparison of ovarian stimulation among three groups ( $\bar{x}$ ± s).

Item	Treatment group (N=38);	Placebo group (N=37);	*P*1 value	Normal group (N=39);	*P*2 value
Days of Gn (days)	10.29 ± 1.72	10.92 ± 1.88	0.114	9.85 ± 1.53	0.027
Total Gn (IU)	3013.16 ± 1005.44	2931.76 ± 822.97	0.688	2195.51 ± 785.40	0.001
E2 on HCG day (pg/mL)	2495.24 ± 1068.46	1555.59 ± 944.127	0.001	3879.74 ± 1109.64	0.001
P (ng/mL) on HCG day	1.256 ± 0.48	1.314 ± 1.02	0.760	1.312 ± 0.62	0.941

P1 value is the treatment group versus placebo group; P2 value is the comparison among three groups

#### 3.1.3 Comparison of ovulation induction results

The number of oocytes retrieved, 2PN fertilization, high-quality embryos and the total number of available embryos in the treatment group were higher than those in the placebo group, and the differences were statistically significant (*P* > 0.05). Although the number of D5 blastocysts was not statistically significant (*P* > 0.05), it also tended to increase. The normal control group was higher than the treatment group and the placebo group in all aspects, and the differences were statistically significant (*P* > 0.01). See **Table 3** for details.

**Table 3:** Comparison of ovulation induction results among three groups ( $\bar{x}$ ± s).

Item	Treatment group (N=38);	Placebo group (N=37);	*P*1 value	Normal group (N=39);	*P*2 value
Number of oocytes retrieved	6.95 ± 2.93	3.76 ± 2.78	0.001	13.72 ± 3.97	0.001
2PN fertilization number	4.61 ± 2.46	2.26 ± 2.06	0.001	10.21 ± 3.92	0.001
D5 Blastocyst Number	0.05 ± 0.23	0.03 ± 0.16	0.900	0.67 ± 1.48	0.002
Number of good quality embryos	0.97 ± 1.31	0.30 ± 0.57	0.045	2.69 ± 2.02	0.001
Total available embryos	2.63 ± 1.87	1.30 ± 1.22	0.041	7.10 ± 4.25	0.001

P1 value is the treatment group versus placebo group; P2 value is the comparison among three groups

#### 3.1.4 Clinical outcome comparison

The clinical pregnancy rate of fresh embryo implantation and frozen embryo implantation in the treatment group was higher than that in the placebo group, but the difference was not statistically significant (*P* > 0.05).The clinical pregnancy rate of frozen embryo implantation in the normal group was higher than that in the treatment group and placebo group, and the difference was statistically significant (*P* > 0.05); the clinical pregnancy rate of fresh embryo implantation was higher than that in the other two groups, but the difference was not statistically significant (*P* > 0.05). Of these, 4 in the treatment group had no embryos available and 10 in the placebo group had no embryos available. See **Table 4** for details

**Table 4:** Comparison of pregnancy outcomes among three groups (%).

Item	Treatment group (N=38);	Placebo group(N=37);	*P*1 value	Normal group(N=39);	*P*2 value
Fresh embryo clinical pregnancy rate (number of fresh embryo clinical pregnancies/number of fresh embryo transfers)	54.55% (6/11)	33.33% (3/9)	0.343	72.73% (8/11)	0.375
Frozen embryo clinical pregnancy rate (number of frozen embryo clinical pregnancies/number of frozen embryo transfers)	34.78% (8/23)	33.33% (6/18)	0.923	71.43% (20/28)	0.009

P1 value is the treatment group versus placebo group; P2 value is the comparison among three groups

### 3.2 Experimental results

In this project, a total of 347 metabolites were targeted and 228 metabolites were identified in the mode of positive and negative mode switching.

#### 3.2.1 Orthogonal partial least squares discriminant analysis (OPLS-DA)

**Figure 1:**
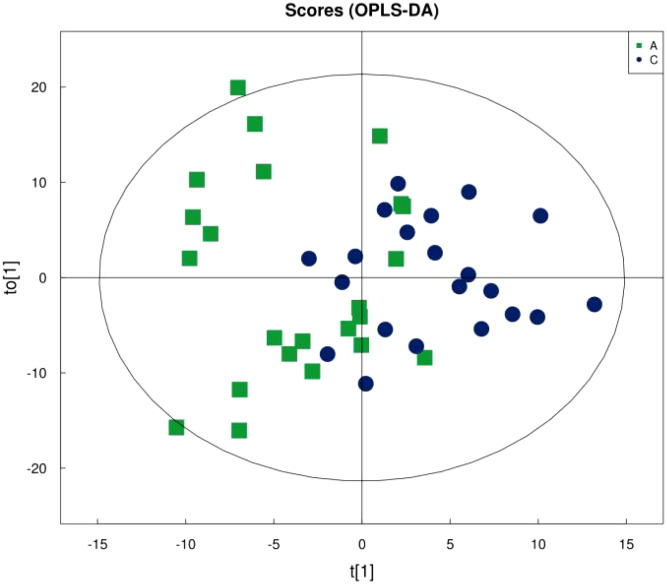
OPLS-DA score plot of treatment group versus normal control group.

**Figure 2:**
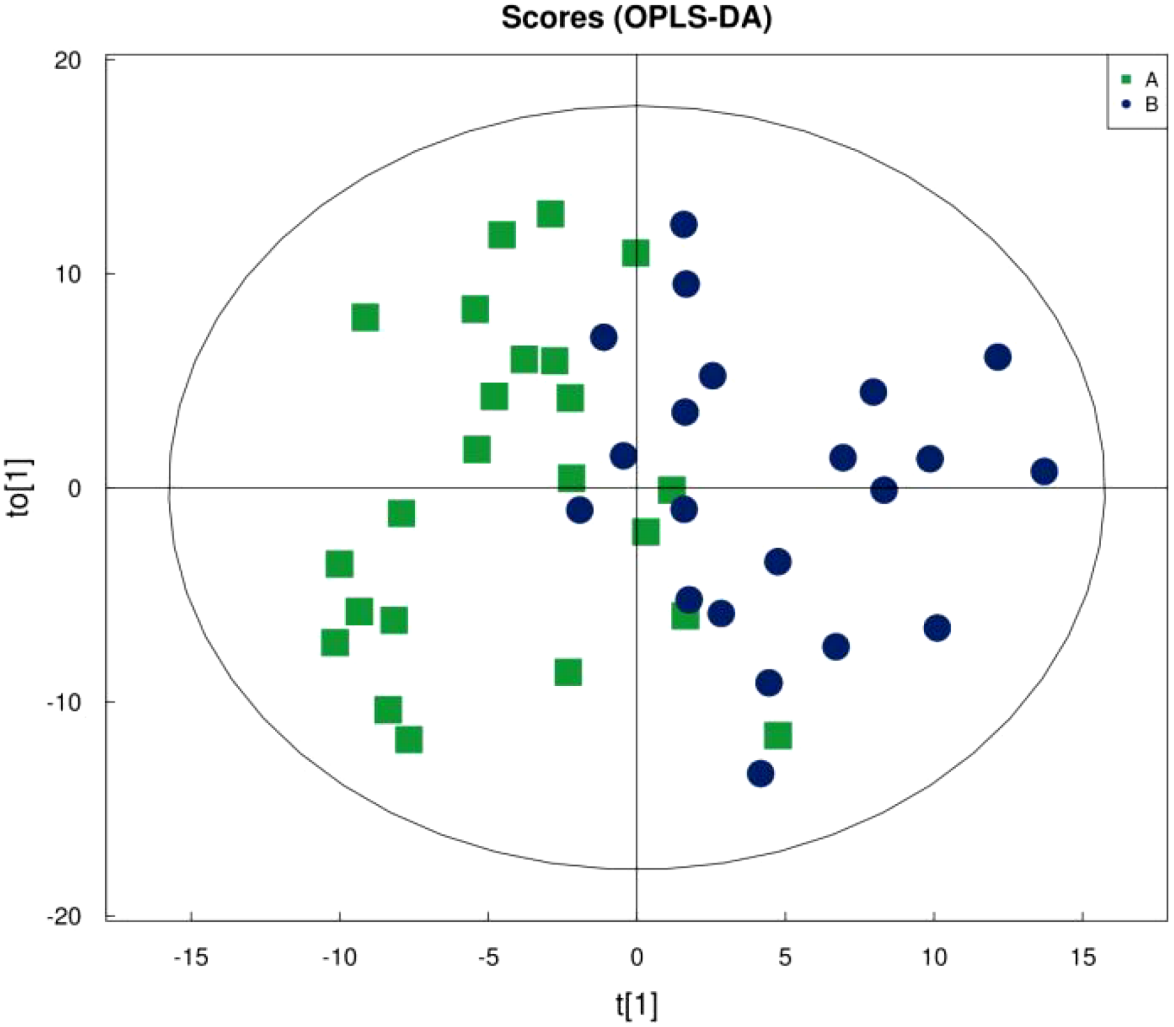
OPLS-DA Score Plot for Treatment Group versus Placebo Group.

**Figure 3:**
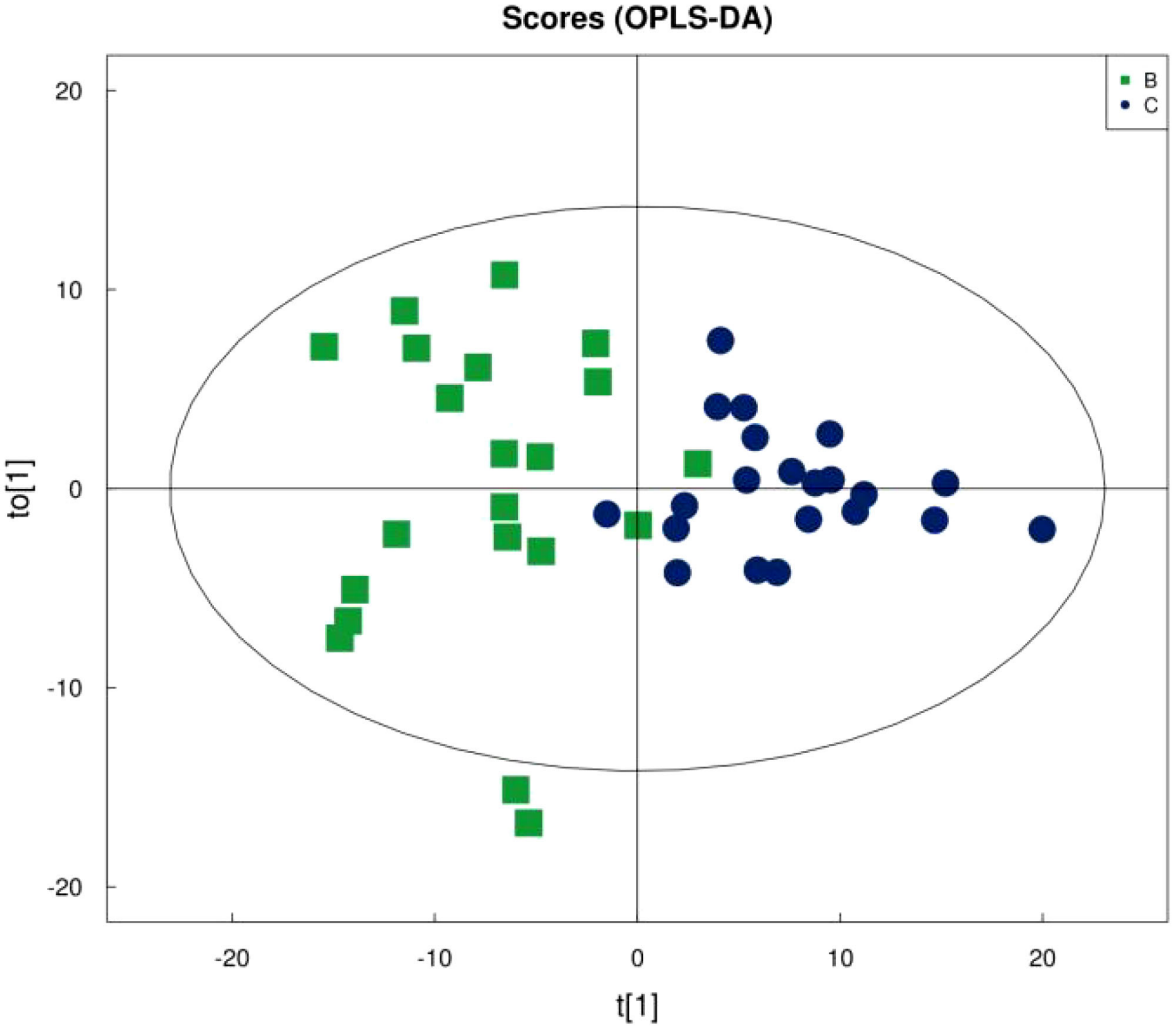
OPLS-DA score plot for placebo vs. normal young adults.

**Figure 1** shows that the samples in the treatment and normal groups clustered after the intervention, **Figure 2** shows that the sample components in the treatment and placebo groups tended to separate, and **Figure 3** shows that the samples in the placebo and normal groups had some separation.

#### 3.2.2 Volcano plot of differential metabolite ion patterns

**Figure 4:**
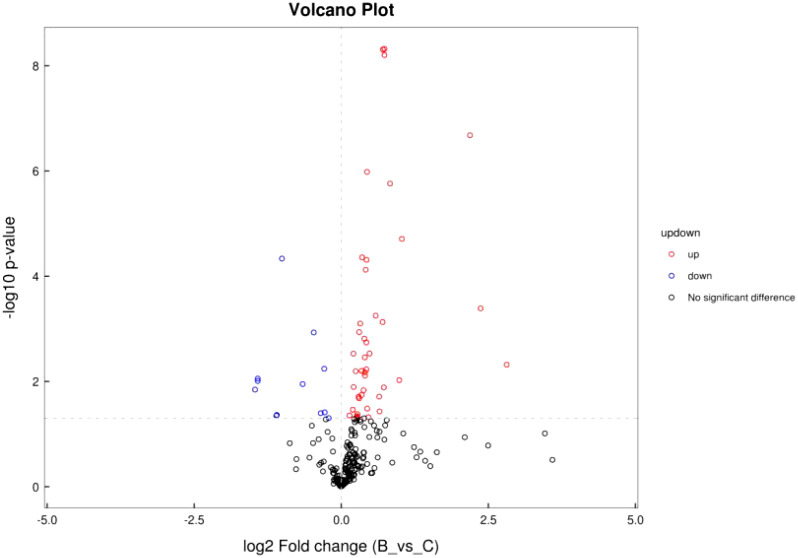
Volcano plot of ion pattern in placebo vs. normal control.

**Figure 5:**
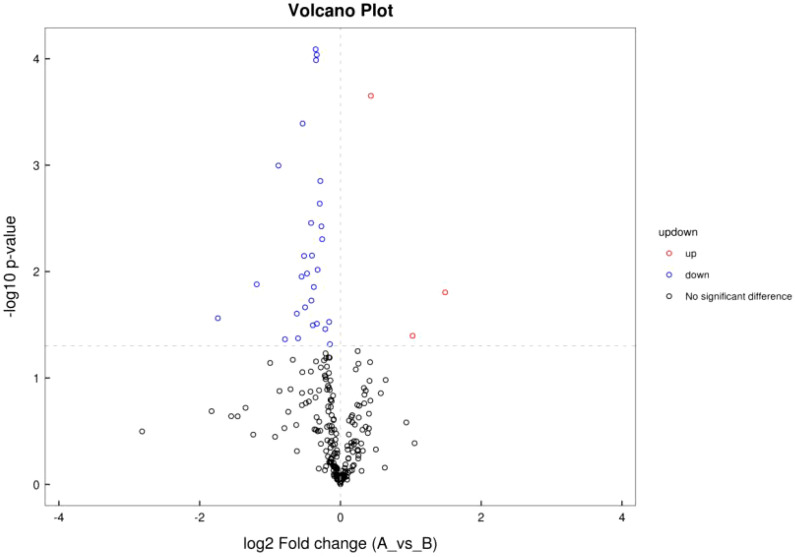
Volcano Plot of Ion Pattern for Treatment vs Placebo.

Differential analysis was performed for all metabolites detected. *P<* 0.05 was used as differential metabolite screening criteria. Volcano plots were used to visualize metabolite changes in older poor ovarian response patients versus normal young women in **Figure 4**, and changes presented in metabolites after treatment with Guilu Erxian ointment in **Figure 5**.

#### 3.2.3 Cluster analysis

**Figure 6:**
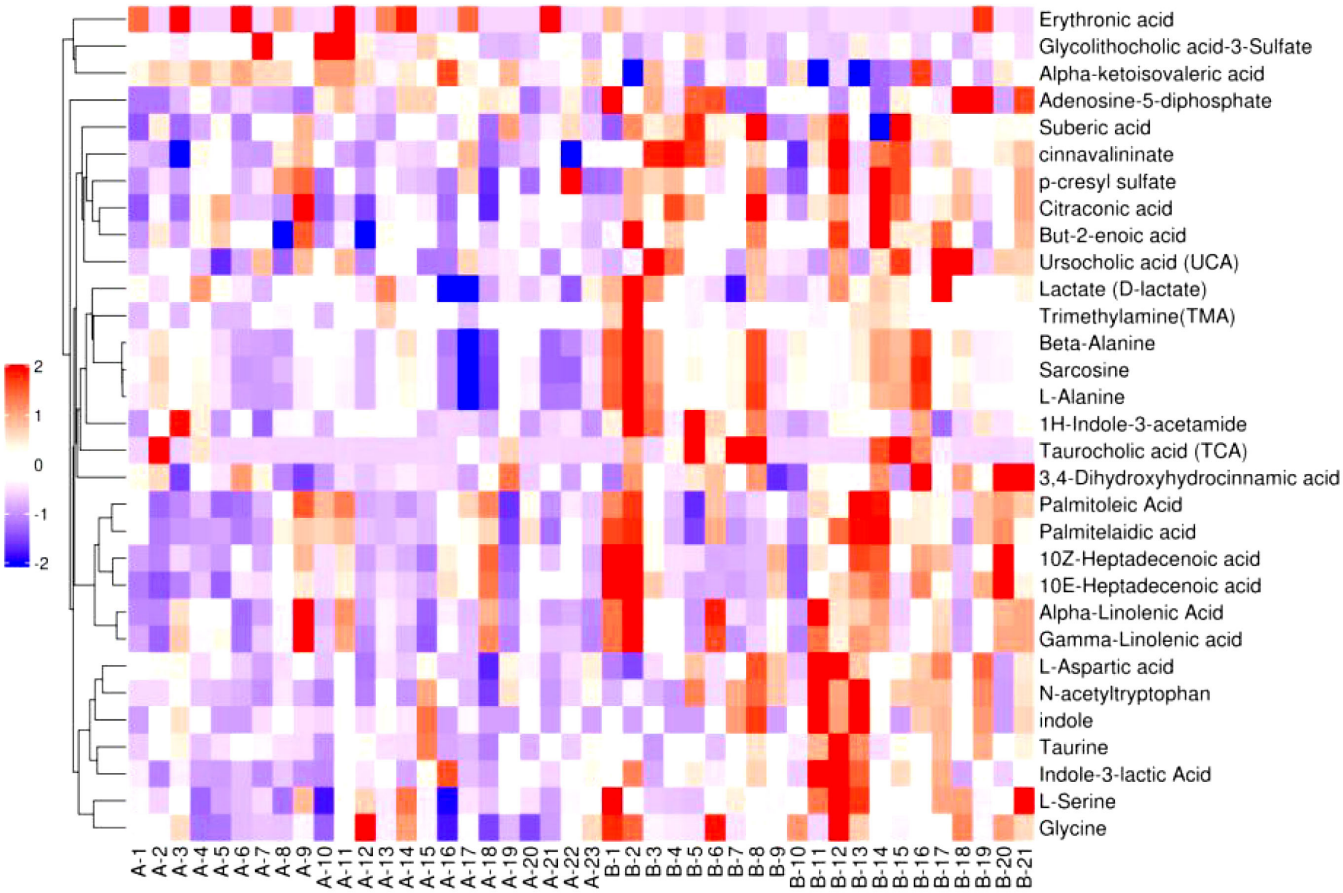
Heatmap of metabolites with significant difference between treatment group and placebo.

**Figure 7:**
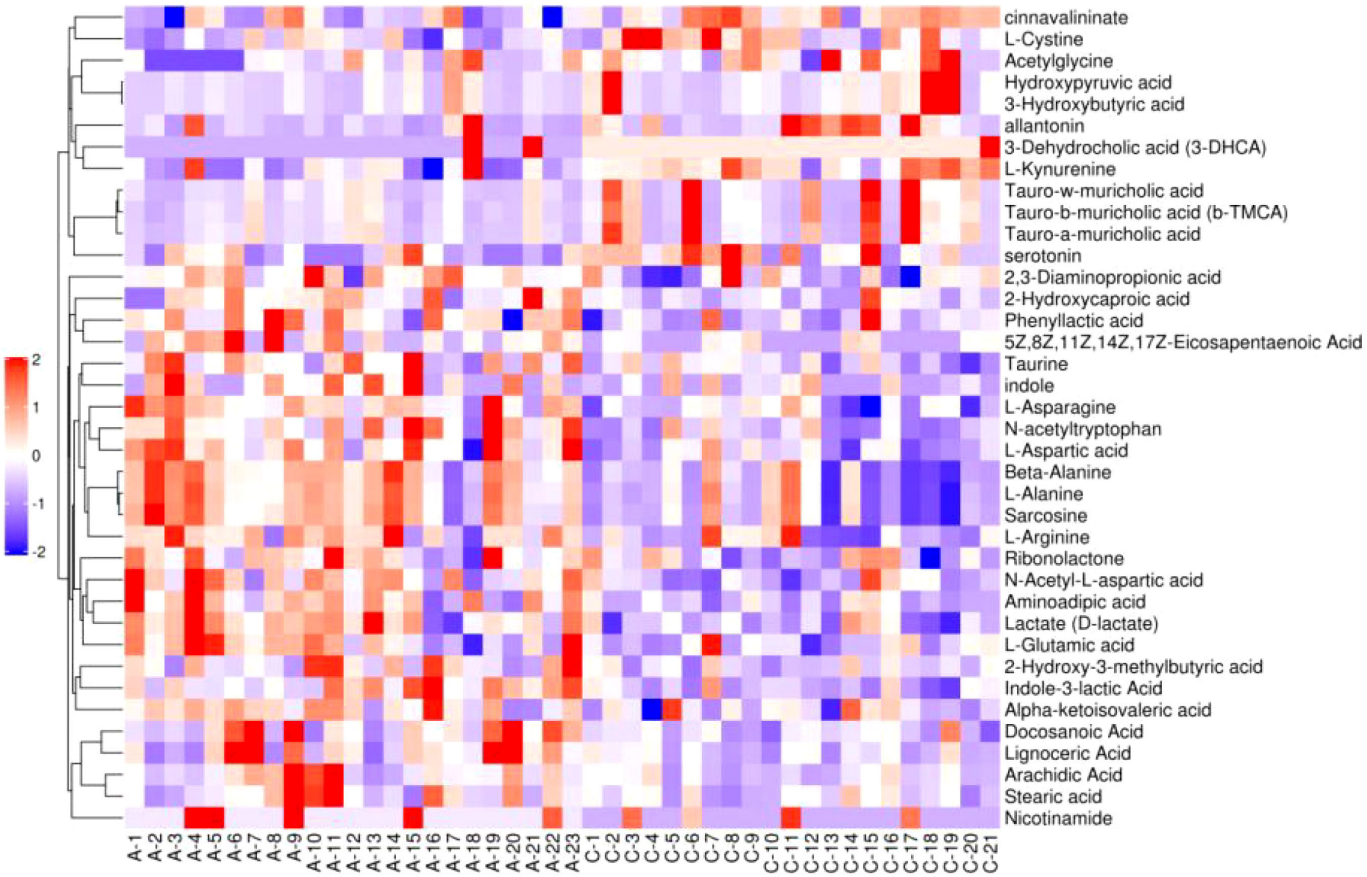
Heatmap of significant differential metabolite hierarchical clustering in treatment group versus normal control group.

**Figure 8:**
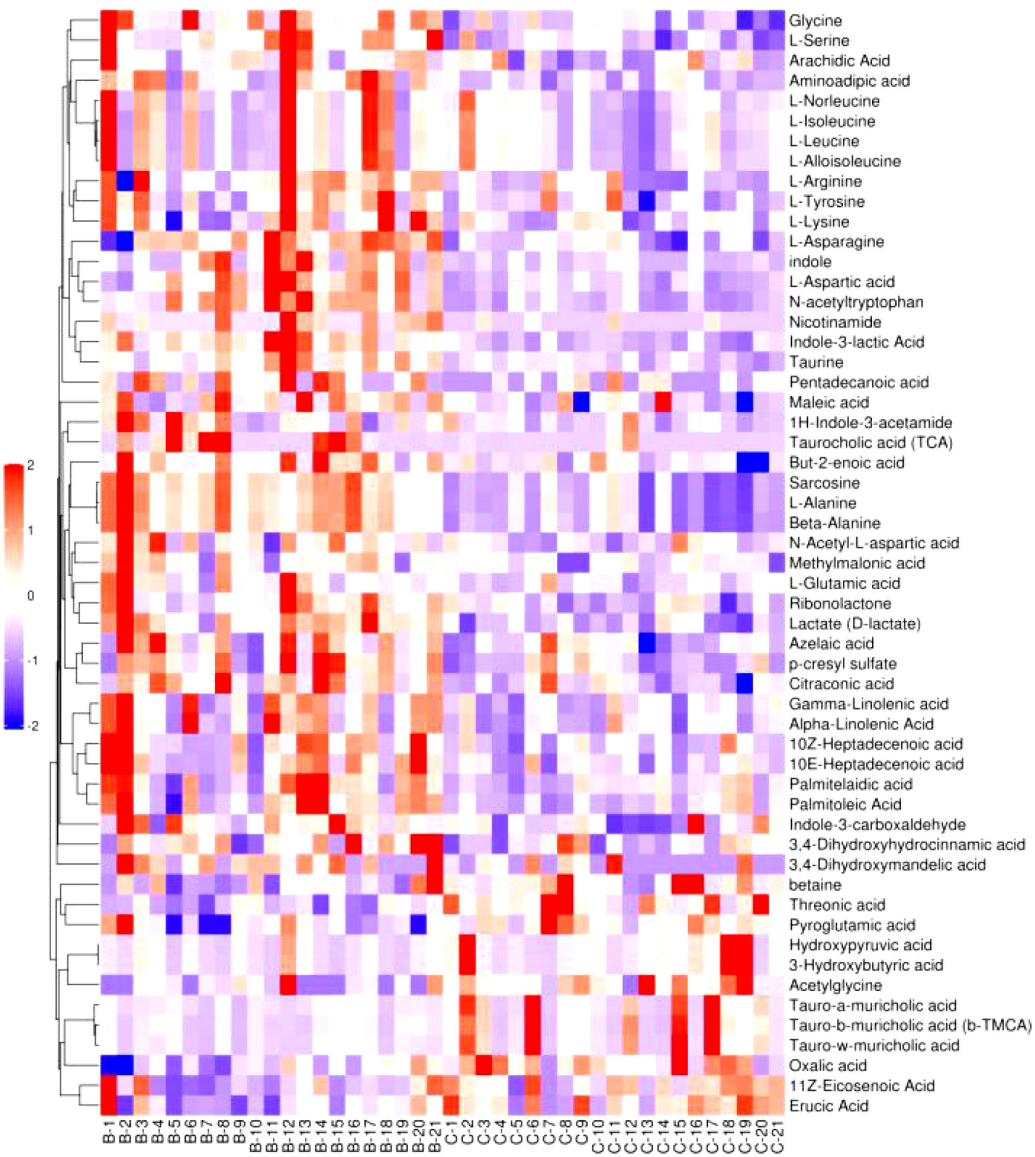
Heatmap of metabolites hierarchically clustered for significant differences between placebo and normal controls.

**Figure 6**–**8** are hierarchical cluster analysis of significantly different metabolites (*P*< 0.05) between the groups. Metabolites clustered within the same cluster have similar expression patterns and may have similar functions or participate in the same metabolic process or cellular pathway together.

#### 3.2.4 ROC curves

**Figure 9:**
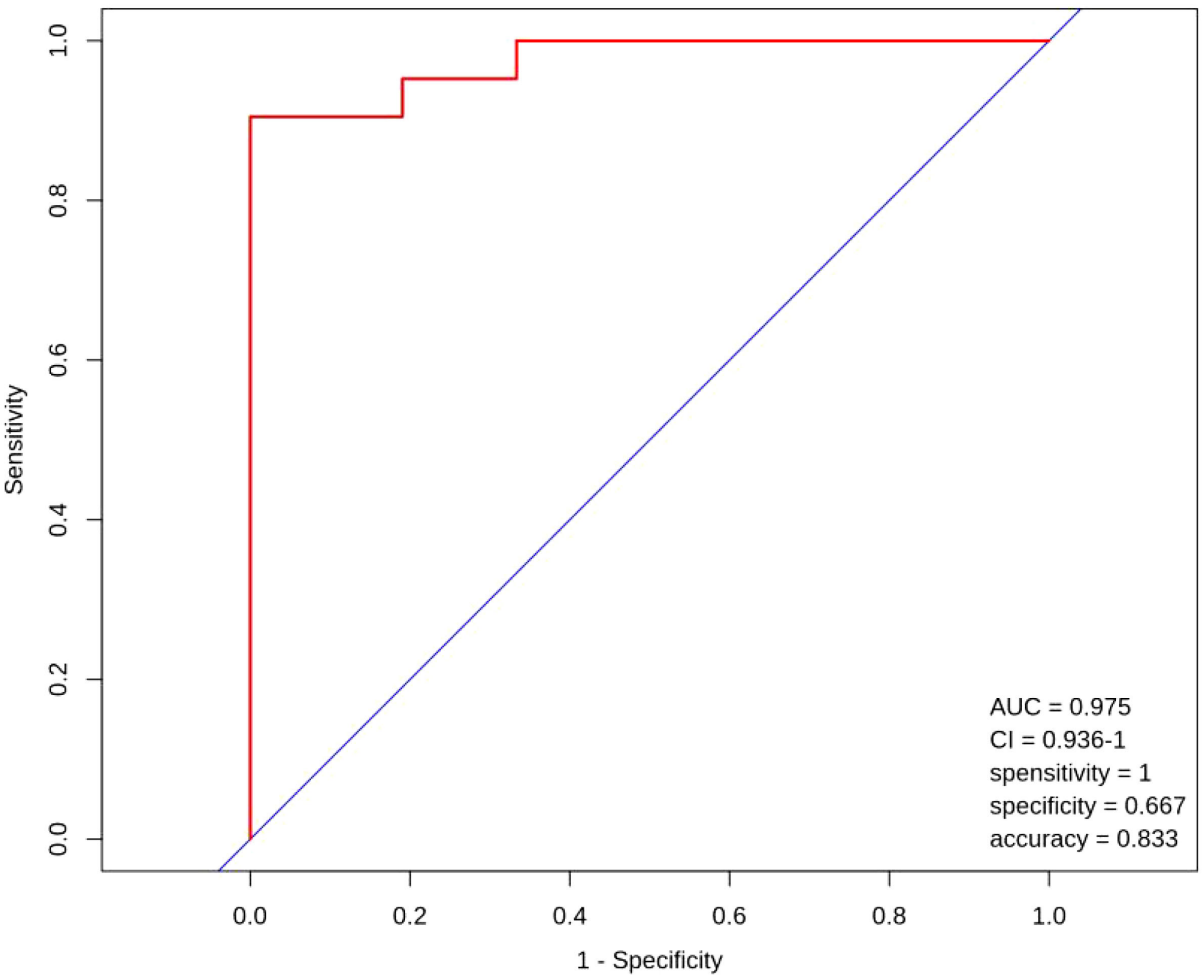
ROC curves.

**Figure 9** shows the ROC curves for the nine metabolites of L-Aspartic acid, L-Alanine, Aminoadipic acid, L-Asparagine, L-Arginine, L-Serine, Gamma- Linolenic acid, Pentadecanoic acid and Alpha-Linolenic Acid with poor ovarian response in advanced age.

#### 3.2.5 KEGG pathway annotation and analysis

**Figure 10:**
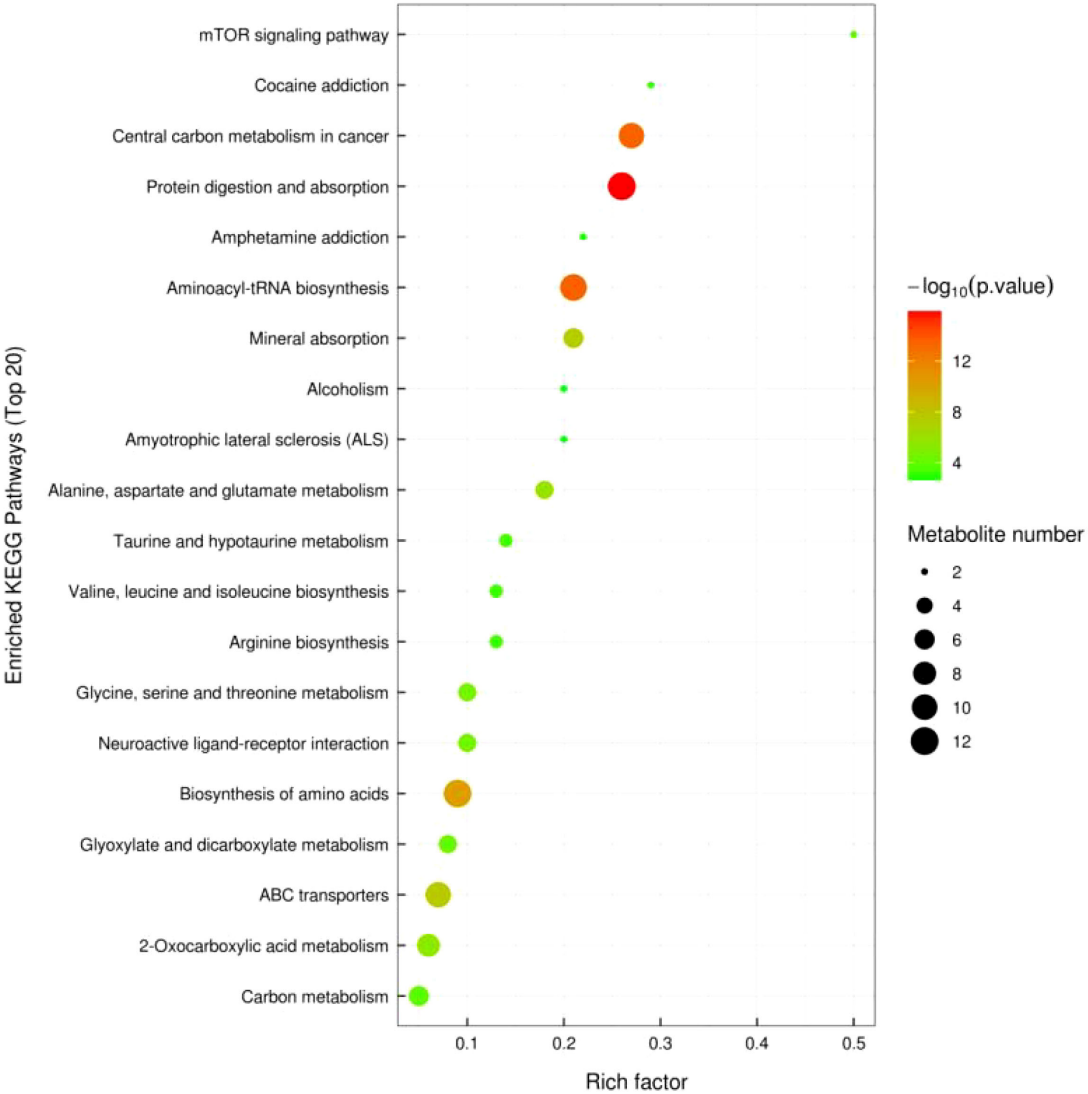
KEGG Enriched Pathway Diagram for Placebo vs. Normal Young Group (Bubble Diagram).

**Figure 11:**
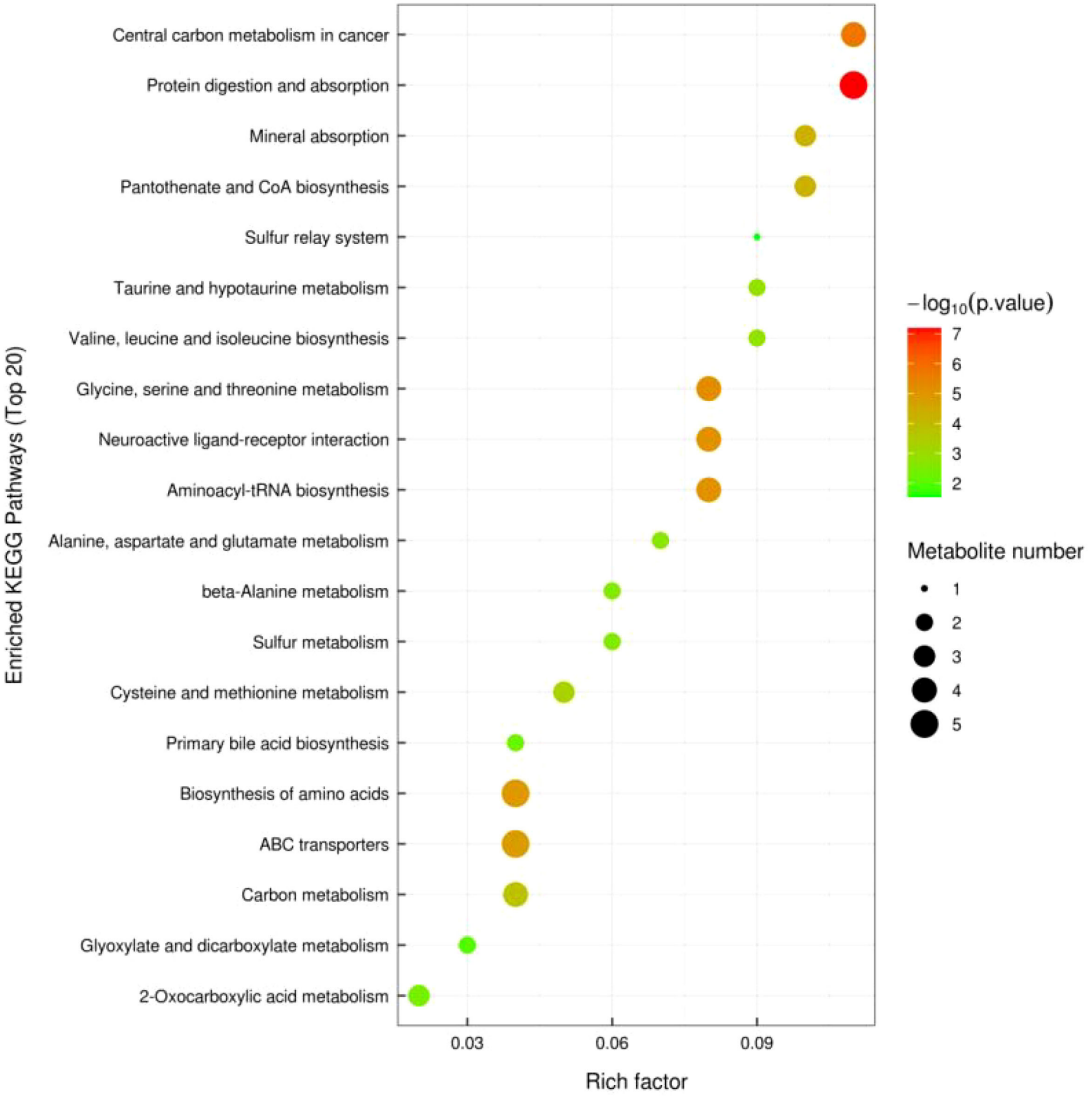
KEGG Enriched Pathway Plot (Bubble Plot) for Treatment vs Placebo.

**Figure 10** shows significant metabolic pathways in older low-responding women versus normal women, and **Figure 11** shows changes in major metabolic pathways after intervention with Guilu Erxian ointment.

## 4 Discussion

Older women with poor ovarian response due to kidney-qi deficiency have decreased ovarian function and ovum mass, decreased oxidative stress and DNA repair damage in the body with age, and changes in autophagy and glycosylation levels, which are accompanied by aging-induced conditions that may cause inflammatory responses in neutrophils. Modern pharmacological studies have found that the traditional Chinese medicine components in Guilu Erxian ointment can improve the above disorders. Tortoise shell glue alleviates renal histopathological changes and has antioxidant activity in rats with kidney yin deficiency to some extent (10). Turtle shell glue can regulate the intrauterine environment by improving hemorheology and regulating E_2_, FSH and LH (11). Wolfberry fruiti can increase antioxidant activity by stimulating endogenous factors in the human body, but also increase the activity of antioxidant enzymes, reduce the occurrence of lipid peroxidation, protect hepatocyte membranes, and thus improve body energy reserve, and aqueous extract of Wolfberry fruiti can also attenuate neuronal cell death induced by homocysteine, thus exerting anti-aging effects (12). Studies have found that kidney-invigorating herbs can improve the clinical outcome of *in vitro* fertilization-embryo transfer by improving the expression of E_2_ receptor in the endometrium, inhibiting oxidative stress in the endoplasmic reticulum, and improving endometrial receptivity (13), while the compatibility of turtle shell and staghorn with ginseng in this prescription can significantly increase the serum estradiol concentration level. The chemical constituents of Fructus Corni mainly contain iridoids and their glycosides, triterpenes, flavonoids, tannins, organic acids, polysaccharides, etc., of which iridoids are not only characteristic components in Fructus Corni, but also have anti-inflammatory, antioxidant and anti-aging effects, and the mRNA levels of CYP1A1, CYP2A4, CYP2A5, CYP3A41 and other genes in liver microsomes of Fructus Corni at different concentrations were significantly increased (14). Crataegus, in some *in vitro* studies, various hawthorn extracts from hawthorn fruit, hawthorn leaves, or hawthorn flowers have antioxidant activity, which may be related to oligomeric proanthocyanidins and total flavonoids, because oligomeric proanthocyanidins and total flavonoids alone have strong inhibitory effects on human low-density lipoprotein (LDL). In various *in vivo* studies, ethanolic extracts were found to protect against oxidative stress in rats with experimental atherosclerosis. This extract prevented the increase of lipid peroxidation and decreased glutathione content and α-tocopherol content in liver, aorta and heart tissues. It also normalized the levels of antioxidant enzymes and increased the activity of superoxide dismutase (SOD) in liver, aorta and heart tissues of hyperlipidemic mice (15). Jujube also has antioxidant effects (16). Therefore, Guilu Erxian ointment can regulate the Hypothalamus-Pituitary-Ovarian axis of the human body and improve the endocrine status of women through the effect of tonifying the kidney and benefiting the essence, and most of the traditional Chinese medicine components such as tortoise shell glue, wolfberry fruit, and evodia rutaecarpa in the formula have antioxidant effects, thereby reducing the production of free radicals, enhancing the phagocytosis of phagocytes, and improving ovarian function through anti-inflammatory and anti-aging functions, thereby improving the fertility decline of older low-responsive women, increasing the number of oocytes retrieved, improving follicular quality and embryo quality, and improving IVF clinical outcomes.

In this study, 228 metabolites were identified in the follicular fluid of older patients with poor ovarian response of kidney-qi deficiency type and normal women, and 55 differential metabolites were found, of which 43 were up-regulated: L-Aspartic acid, L-Alanine, Sarcanine, Beta-Alosine, Aminoadipic acid, L-Asparagine, L-Arginine, L-Serine, etc. There were 12 kinds of downward adjustments: Pyroglutamic acid, Acetylglycine, 11Z-Eicosenoic Acid, Erucic Acid, etc. It can be divided into seven categories, which are amino acids, fatty acids, bile acids, indoles, hydroxyl acids, binary carboxylic acids, and others, mainly involving metabolic pathways such as Protein metabolism and absorption, Glycine, serine and threonine metabolism acids, Biosynthesis of amino acids digestion, Nicotinate and nicotinamide. Combined with previous studies that found a large correlation between L-aspartic acid, Alanine, Aminoadipic acid, L-asparagine, Arginine, L-serine, γ-linolenic acid, pentadecanoic acid, α-linolenic acid and poor ovarian response in advanced age, we speculated that these substances may be biomarkers for poor ovarian response in advanced age patients with kidney qi deficiency.

L-Aspartic acid is a non-essential amino acid. In 2020, Ichikawa (17) found that L-aspartic acid is required to convert citrulline to L-arginine, and L-aspartic acid supplementation may help improve endothelial function. In 2021, metabolomics analysis and quantitative pathway analysis showed that L-aspartate was significantly increased in nicotinic acid and nicotinamide metabolic pathways in women with poor ovarian response (18), which was consistent with our study, so L-aspartate may be related to ovarian function.

The 2013 study demonstrated that indirectly confirmed its role in the pathogenesis of premature ovarian failure in women by replacing positively selected amino acids with L-Alanine or the most representative residue in other investigated species is important for the normal function of the protein as well as fertility in women (19). In 2017, it was found that women have a high concentration of alanine in the uterus on the fourth day after ovulation, and its accumulation in the endometrium may be important for endometrial cell function (e.g. proliferation), and the transport and metabolism pathways of alanine directly provide the endometrial demand for growth and function to ensure endometrial receptivity (20).

Aminoadipic acid accumulates in large amounts with aging (21). α-Aminoadipic acid semialdehyde (α-AS) is the main protein carbonyl and intermediate oxidation product of lysine, which is further oxidized to α-aminoadipic acid (α-AA) in the presence of peroxide. α-AA is considered a reliable indicator of protein oxidation, aging, and disease in the human body. α-AA has been shown to be a more reliable marker of protein oxidation, rather than a precursor of protein oxidation, under a variety of pathological conditions such as skin aging, crystal sclerosis, diabetes, and renal failure (21–25). Under conditions of oxidative stress, proteins in body tissues and fluids react, leading to their oxidation and glycation, and reactive oxygen species (ROS) generated during metabolic activities caused by oxidative stress are one of the most important contributing factors to ovarian aging (26, 27). It can therefore be inferred that aminoadipic acid may play a role in the pathogenesis of decreased ovarian reserve in addition to being an indicator of oxidative stress and physiological damage.

Previous studies have found significant differences between L-Asparagine in endometriosis and malignancy (28, 29), and it has been demonstrated that asparagine, in studies of women undergoing *in vitro* fertilization (IVF), is considered a biomarker of reduced hormone sensitivity in controlled ovarian hyperstimulation before IVF, which reduces hormone sensitivity, promotes better follicular growth, and increases the chance of pregnancy Improving clinical pregnancy rates versus live birth rates (30–37), L-asparagine was significantly different in our study, thus infertile patients with advanced age poor ovarian response might be inferred to L-asparagine as a biomarker.

M-Arginine is a non-essential amino acid that plays a critical role in various biological processes including immune responses (38–40). POF1B may act as an anti-apoptotic factor and can slow down the process of germ cell loss, loss of POF1B function may lead to excessive germ cell apoptosis and POF, POF1B is highly homologous to the myosin tail of human myosin and is a protein involved in actin filament interaction, with lack of phosphorylation at the arginine site, which leads to loss of POF1B protein function (41, 42). Goto E et al. found that prenatal oral l-arginine had favorable effects on IUGR neonates, preterm birth, RDS, birth weight, and gestational age in women with a history of adverse pregnancy outcomes (43). Cieri-Hutcherson et al. demonstrated through a meta-analysis that L-arginine can be used to treat active sexual desire disorder (HSDD) or related disorders in women with HSDD (44).

As one of the most important signaling pathways, the Hippo signaling pathway plays an important role in regulating female reproductive system development (45–47). Yuan Cheng and colleagues first demonstrated the ability of actin polymerization-promoting drugs to inhibit the Hippo signaling pathway and promote organ growth, and showed that disruption of Hippo signaling in ovarian follicles can be used for infertility treatment, and in mammals, the Hippo pathway has four core components, including the serine/threonine kinase MST1/2 (homologue of Hippo/Hpo) (48, 49). YAP is a transcription factor that enters the nucleus following phosphorylation and may play a role in the transition from primordial to primary follicles. High density activates the Hippo pathway and promotes phosphorylation of serine on YAP, leading to its nuclear export, growth inhibition, and even apoptosis (50, 51). It is therefore concluded that L-Serine may affect female fertility by interfering with the Hippo pathway and causing follicular failure in females.

Gamma-Linolenic acid is synthesized from linoleic acid (52), an omega 6 polyunsaturated fatty acid, and animal studies have shown that increases in linoleic acid inhibit expansion of cumulus cells and development of mature oocytes (53). In addition, linoleic acid has been associated with more dysmorphic cumulus oocyte complexes and reduced success following IVF-ICSI in human studies (54, 55). Therefore, combined with this study, it is inferred that lower γ-linolenic acid facilitates oocyte quality, early embryonic development, and successful *in vitro* fertilization.

Among fatty acids, odd-chain saturated fatty acids (mainly pentadecanoic acid and heptadecanoic acid), conjugated linoleic acid, and palmitoleic acid can be acquired from diet, specific metabolic pathways, or intestinal bacteria (56–60). It has been shown that there is an independent positive relationship between the triglyceride percentage of pentadecanoic acid and cleavage rate, and the level of pentadecanoic acid in follicular fluid is significantly correlated with gene expression involved in lipogenesis and fatty acid elongation as well as the number of lipid droplets in cumulus granulosa cells, suggesting that the regulatory role of pentadecanoic acid in follicular lipid metabolism may greatly improve oocyte quality and early embryonic growth through multiple mechanisms, including fatty acid distribution to lipid droplets, thereby providing energy and preventing lipotoxicity, promoting oocyte fluidity and embryonic cell division as regulation of signal transduction and cellular oxidative stress (61–64).

The level of Alpha-Linolenic Acid in follicular fluid is inversely related to the ability of oocytes to develop to metaphase, and researchers have also found that as follicles grow α-linolenic acid levels decrease, suggesting that the latter fatty acid is converted to long-chain counterparts by sequential elongation and desaturation reactions, and have also demonstrated that supplementation of bovine COC with α-linolenic acid eliminates the lipotoxic effects of non-esterified fatty acids on oocyte developmental ability (61). This is in line with our findings, illustrating the negative correlation between α-linolenic acid levels and follicular development.

### 4.1 Amino acid metabolism

Female fertility has a great relationship with the internal environment of the uterus, and hormonal changes can cause changes in the uterus, which are essential for uterine receptivity as well as for embryonic development and implantation. In the first two weeks of embryo transfer, conception of the fetus depends entirely on the intrauterine environment created by endometrial secretions or molecules entering the uterine cavity before implantation and placenta (65). Amino acids are important components of these secretions and play an important role in embryo survival during early pregnancy (66–70). Optimal amounts of essential and non-essential atomic absorption are important for embryonic development, which is altered if the number of these molecules is not ideal (71–73). Regulation of amino acid transport and concentration in cells and tissues, including placenta and endometrium, depends on specific transporters (74). Changes in amino acid transporter transcript profiles reveal changes in amino acid availability in the uterine cavity, and amino acid transporter genes are simultaneously increased in maternal endometrium and embryonic cells. This suggests that amino acids are transported from the maternal circulation to the endometrial cavity for embryonic development.

Changes in the endocrine environment before and after ovulation have been found to affect amino acid transport from maternal circulation to endometrial cells and finally to the uterine cavity through expression of the AA transporter pathway, and concentrations of taurine, alanine, and α-aminobutyric acid are higher in uterine cavity washings on day 4 after ovulation, which represents a hyporeceptive uterus in this animal model (20). Our findings are consistent with the up-regulation of taurine, alanine and α-aminobutyric acid metabolites in the original treatment group compared with the normal group, down-regulation after intervention, and improvement in clinical outcomes compared with the placebo group, so amino acid transport and availability *in utero* may play an important role in maternal and embryonic health.

Differential metabolites in amino acid metabolism pathways also play a role in ovarian development. Arginine, as a precursor of nitric oxide, polyamines and creatine synthesis, has different physiological functions. Most metabolites in the arginine metabolism pathway increased from stage II to stage III, including arginine, proline, γ-aminobutyric acid-related metabolites, and creatine related metabolites, suggesting that enhanced arginine metabolism is a biomarker for stage III ovaries and is used to control cell proliferation and differentiation during early ovarian development. In addition, as a cellular energy shuttle (phosphocreatine can regenerate ATP), creatine plays a critical role in regulating energy metabolism and may be a metabolic response to the high energy requirements of the developing ovary (75). The results of this study showed that amino acids such as arginine, creatine and aminobutyric acid changed before and after intervention, and some showed down-regulation, which may affect ovarian development and hormone level changes by regulating energy metabolism, and then affect follicular growth and embryonic development.

Amino acids act in various ways as organic osmotic cells, intracellular buffers, heavy metal chelators and energy substrates (76). Amino acid transporters have been detected in mouse oocytes and cumulus cells and mediate uptake of amino acids by oocytes *via* gap junctions (77). It has been found that the most abundant amino acids in the follicular fluid of pigs are glycine, glutamate, and alanine, which are at least twice as concentrated as other amino acids, and most amino acids, except aspartate, are less concentrated in large follicles than in small follicles, and amino acids added to IVM medium do not have any stimulating effect on nuclear maturation, but increase embryo formation after fertilization (78). This is basically consistent with our study that there are fewer mature follicles in older women with low prognosis, and the concentrations of glycine, glutamate, and alanine in follicular fluid are higher than those in the normal young group, which are down-regulated after intervention with kidney-invigorating herbs, thus indicating that amino acids have a beneficial effect in oocyte maturation and may be in cytoplasmic maturation. However, it is unclear in this study whether the increase in embryonic development is due to a specific effect of amino acids on cytoplasmic maturation or to a protective effect of amino acids against culture-induced stress, and further studies are needed to determine the exact role of specific amino acids in IVF.

### 4.2 Fatty acid metabolism

The concentration of fatty acids in the follicular microenvironment or oocyte culture medium is associated with quality and development during *in vitro* fertilization and is also an important substrate for oocyte maturation (79–82).Fatty acids are divided into three major classes: saturated fatty acids (SFA), monounsaturated fatty acids (MUFA), and polyunsaturated fatty acids (PUFA), which correspond to molecules without, one, or more double bonds in their acyl chains, respectively. In addition, polyunsaturated fatty acids are divided into omega-3 (n-3), omega-6 (n-6), and omega-9 (n-9) fatty acids based on the proximity of the first double bond to the terminal methyl group (1 carbon).SFA and MUFA are mainly used by oocytes and surrounding cumulus cells to produce energy and structural elements (83, 84), while polyunsaturated fatty acids play a wide range of roles in oocyte biology and metabolism. Cumulus cells nourish developing oocytes through gap junctions between cells. Gap junctions allow bidirectional trafficking of small metabolites, contributing to oocyte maturation and acquisition of developmental competence (85, 86).The cumulus cell layer is the first metabolic region affected by altered levels of free fatty acids in follicular fluid. Indeed, exposure to saturated fatty acids *in vitro* induces apoptosis in cumulus cells but not in oocytes (87, 88).Oocytes surrounded by the cumulus cell layer are able to bind fatty acids in the medium, and oocytes can store fatty acids as neutral lipids into lipid droplets. A hallmark of this storage is an increase in the size and number of lipid droplets in oocytes upon exposure to unsaturated free fatty acids (89).It has been postulated that after fertilization, stored neutral lipids are important as energy sources and precursors for *de novo* membrane synthesis during early embryonic development (90–92).Increased concentrations of free fatty acids in follicular fluid may alter the lipid storage properties of oocytes and thus adversely affect the later developmental competence of oocytes.

A total of 31 metabolites were significantly changed in the treatment group compared with the placebo group after conditioning with kidney-invigorating herbs, including 3 up-regulated and 28 down-regulated metabolites. A total of 20 metabolic pathways were found to be significantly changed by KEGG pathway analysis. After comprehensive comparison of the differential metabolites among the three groups, we found that the nine metabolites, L-aspartate, glycine, L-serine, palmitoleic acid, palmitate, alanine, γ-linolenic acid, α-linolenic acid, and N-acetyltryptophan, were the most correlated, and they all had one property: Guilu Erxian Ointment, a kidney-invigorating Chinese medicine, changed from the original up-regulation to down-regulation after intervention. Combined with previous studies and this differential metabolite profile, we found that the two metabolic pathways of amino acid metabolism and fatty acids were mainly related to the mechanism by which kidney-invigorating herbs improved IVF outcomes in older patients with poor ovarian response of kidney-qi deficiency type.

## Data availability statement

The raw data supporting the conclusions of this article will be made available by the authors, without undue reservation.

## Ethics statement

The studies involving human participants were reviewed and approved by ethics committee of Reproductive Medicine, Affiliated Hospital of Shandong University of Traditional Chinese Medicine. The patients/participants provided their written informed consent to participate in this study.

## Author contributions

SZ and SJ designed and supervised this study. MY wrote the manuscript. CX and SJ modified the manuscript. MY made significant contributions to the sample size estimation and performed the statistical analysis. All authors contributed to the article and approved the submitted version.
